# Discovery of active enhancers through bidirectional expression of short transcripts

**DOI:** 10.1186/gb-2011-12-11-r113

**Published:** 2011-11-14

**Authors:** Michael F Melgar, Francis S Collins, Praveen Sethupathy

**Affiliations:** 1 Genome Technology Branch, National Human Genome Research Institute, National Institutes of Health, 9000 Rockville Pike, Bethesda, MD 20892, USA; 2 Current address: School of Medicine, The University of California at San Francisco, 505 Parnassus Avenue, San Francisco, CA 94143, USA; 3 Department of Genetics, The University of North Carolina at Chapel Hill, 130 South Building, Chapel Hill, NC 27599, USA; 4 Carolina Center for Genome Sciences, The University of North Carolina at Chapel Hill, 130 South Building, Chapel Hill, NC 27599, USA; 5 Lineberger Comprehensive Cancer Center, The University of North Carolina at Chapel Hill, 130 South Building, Chapel Hill, NC 27599, USA

## Abstract

**Background:**

Long-range regulatory elements, such as enhancers, exert substantial control over tissue-specific gene expression patterns. Genome-wide discovery of functional enhancers in different cell types is important for our understanding of genome function as well as human disease etiology.

**Results:**

In this study, we developed an *in silico *approach to model the previously reported phenomenon of transcriptional pausing, accompanied by divergent transcription, at active promoters. We then used this model for large-scale prediction of non-promoter-associated bidirectional expression of short transcripts. Our predictions were significantly enriched for DNase hypersensitive sites, histone H3 lysine 27 acetylation (H3K27ac), and other chromatin marks associated with active rather than poised or repressed enhancers. We also detected modest bidirectional expression at binding sites of the CCCTC-factor (CTCF) genome-wide, particularly those that overlap H3K27ac.

**Conclusions:**

Our findings indicate that the signature of bidirectional expression of short transcripts, learned from promoter-proximal transcriptional pausing, can be used to predict active long-range regulatory elements genome-wide, likely due in part to specific association of RNA polymerase with enhancer regions.

## Background

Cellular identity and function are defined in large part by regulatory networks that determine gene expression profiles. Control of gene expression is complex, multi-faceted, and coordinated [1,2]. Over the past decade, with the advent of high-throughput genomic technologies, many systems-level biological approaches have been developed to help resolve these complexities, although substantive questions remain [3,4]. Recent large-scale human genetic studies have revealed that most complex disease-associated variants map to within non-coding genomic regions [5-7], providing additional impetus to expand current catalogs of gene regulatory elements and better understand cellular control of gene expression.

The first step in gene expression is the recruitment to gene promoters of a multi-protein transcription initiation complex [8], which includes RNA polymerase (RNAP). Once RNAP is stably bound to the template DNA, it becomes transcriptionally engaged, and commences elongation. It was noted over two decades ago that RNAP could pause/stall at promoters, waiting for a specific signal to continue productive transcription [9]. However, this type of regulation of transcriptional elongation was thought to be an atypical phenomenon. Three recent genome-scale approaches, employing high-throughput sequencing technologies, have revealed that promoter-proximal RNAP pausing is widespread, and likely a common mode of gene regulation [10-12].

One of these methods, global nuclear run-on followed by high-throughput sequencing (GRO-seq), provides a density map of transcriptionally engaged RNAP across the genome by purifying, sequencing, and mapping nascent RNAs [10]. When applied to human lung fibroblasts (IMR90), GRO-seq revealed that promoter-proximal pausing is almost always accompanied by short, divergent (anti-sense) transcription [10]; hereafter this signature is referred to as bidirectional expression of short transcripts (BEST). Two independent methods confirmed this signature in both murine embryonic stem cells [13] and HeLa cells [14], suggesting that BEST is a general feature of RNAP pausing in mammalian tissues.

The functional consequences of promoter-proximal RNAP pausing are likely diverse [15-17]. One recent study found that paused RNAP facilitates the induction and maintenance of an open chromatin conformation near a gene promoter [18]. We reasoned that RNAP pausing, and thus BEST, may occur at specific non-promoter regions where open chromatin is present. Indeed, studies in yeast have shown that pausing also occurs at certain non-promoter sites within gene bodies [12,19,20].

In this study, we first sought to assess whether BEST is a common feature in human cells. We analyzed published IMR90 GRO-seq data [10] to define actively transcribed genes and used the GRO-seq data at the promoters of these genes for supervised training of a Naïve Bayes classifier (NBC). Using the NBC, we predicted nearly ten thousand high-confidence, non-promoter-associated BEST events genome-wide. Intriguingly, BEST significantly co-occurred with open chromatin loci (DNase hypersensitivity sites (DHSs) [21]), and was even more strongly associated with DHSs that overlap regions enriched for histone H3 lysine 27 acetylation (H3K27ac), the most reliable chromatin marker to date of active enhancers [22,23]. BEST was modest at regions bound by the CCCTC-binding factor (CTCF), which serve as either direct transcriptional modulators or insulators depending on chromatin context [24,25]. Further analysis of epigenomic data revealed that several active chromatin marks, including histone H3 lysine 18 acetylation (H3K18ac) and histone H4 lysine 5 acetylation (H4K5ac), but not H3 lysine 4 tri-methylation (H3K4me3), were significantly enriched at non-promoter-associated BEST loci relative to background expectation. Overall, our findings indicate that BEST can demarcate active non-promoter regulatory elements, likely due in part to the specific association of RNAP with distal regulatory elements.

## Results

**Figure 1:**
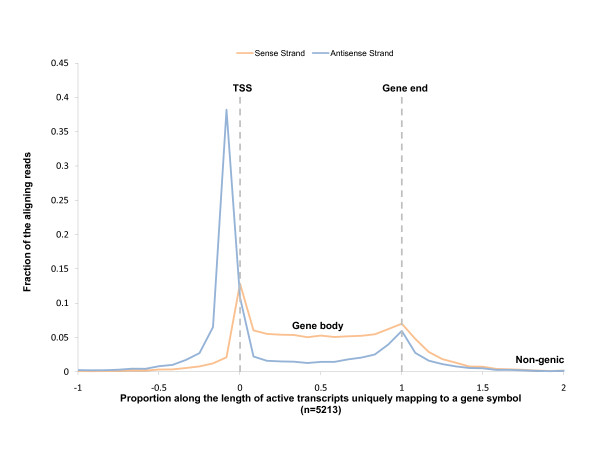
**IMR90 GRO-seq read distribution along the length of actively transcribed genes**. As reported previously [10], a significant spike in both sense (orange) and anti-sense (blue) reads is observed near the transcription start site (position 0 - TSS). A smaller spike is evident at the annotated gene end (position 1 - Gene end). As expected, very little GRO-seq signal is observed in non-genic regions (positions less than -0.5 and greater than 1.5), and RNA polymerase-mediated transcription continues past the annotated gene end (positions between 1 and 1.5).

To confirm BEST at active promoters, we first sought to define from IMR90 GRO-seq data a set of transcriptionally active genes (Materials and methods). We calculated the average reads per kilobase normalized for mapability for every known human gene longer than 3 kb, and performed a receiver operating characteristic (ROC) analysis using 1,522 expressed genes and 2,046 non-expressed genes from an IMR90 microarray expression dataset [26]. The most accurate cutoff for transcriptional activity was determined to be 5 reads/kb/mapability (Additional file 1), yielding 14,145 active RefSeq transcripts, of which 5,213 uniquely mapped to a gene symbol. We computed the average GRO-seq signal along the length of all 5,213 transcripts, and confirmed the previously reported enrichment of sense and antisense reads near the transcription start site (TSS) [10] (Figure 1), which is characteristic of mammalian transcriptional pausing. We also observed a similar, but substantially dampened, pausing signal at the gene end (Figure 1). As shown by Core *et al. *[10], although the promoter-proximal pausing index [27,28] was inversely correlated with gene transcription (Additional file 2), pausing was still detectable at the promoters of very highly expressed genes.

### A probabilistic model predicts nearly 10,000 non-promoter-associated BEST loci genome-wide in human cells

**Figure 2:**
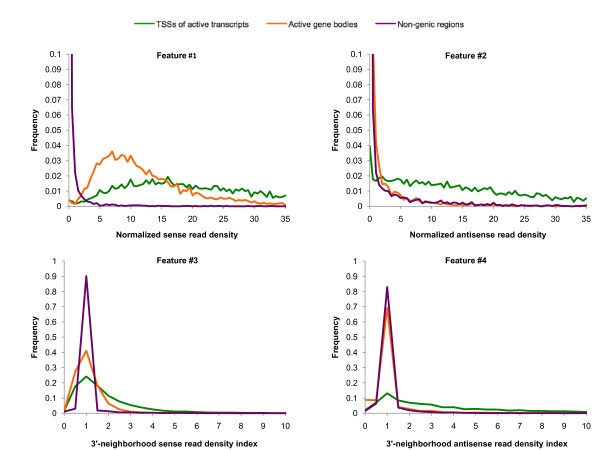
**Probability distributions for four of the six features used to train the BEST predictor**. Probability distributions are shown for each of four features in non-transcribed (purple), active transcription start sites (TSSs; green), and transcriptional elongation states (orange). Feature #1: normalized sense strand reads density (reads/kb/mapability). Feature #2: normalized antisense strand read density (reads/kb/mapability). Feature #3: ratio of the normalized sense strand read density to that in a 2-kb window immediately 3' of the test window. Feature #4: ratio of the normalized antisense strand read density to that in a 2-kb window just 3' of the test window. (Not shown are feature #5, ratio of the normalized sense strand read density to that in a 2-kb window immediately 5' of the test window, and feature #6, ratio of the normalized antisense strand read density to that in a 2-kb window just 5' of the test window.)

**Figure 3:**
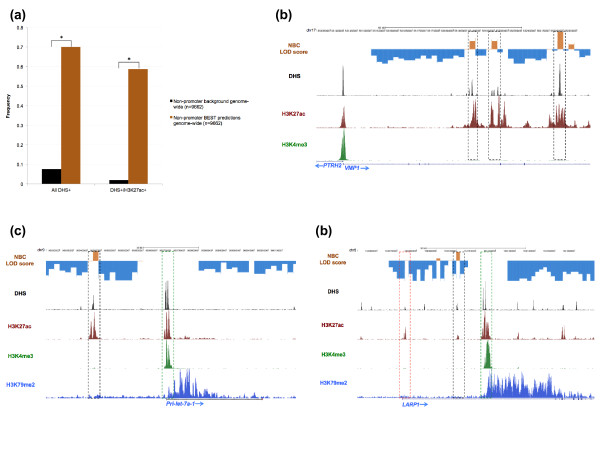
**Overlap among non-promoter-associated BEST predictions, DNase hypersensitive sites, and H3K27ac peaks in IMR90 cells**. **(a) **The frequency of overlap (y-axis) is shown between BEST predictions (brown) and DHSs (x-axis; All DHS+), and DHSs that overlap H3K27ac peaks (x-axis; DHS+/H3K27ac+), relative to background expectation (black). Background regions are non-promoter-associated 2-kb windows randomly selected from the genome. *P*-values were calculated using the two-tailed chi-squared test; *Chi-squared test *P*-value < 0.00001. **(b-d) **Overlap between BEST predictions with LOD score > 2.5, DHSs, and H3K27ac peaks, in IMR90 cells is shown at three separate loci: (b) vacuole membrane protein 1 (*VMP1*) - non-promoter-associated BEST loci (black dashed boxes) are enriched for DHSs and H3K27ac, and depleted of H3K4me3; (c) primary transcript of microRNA let-7a-1 (*Pri-let-7a-1*) - a BEST locus (black dashed box) upstream of the promoter (green dashed box) lacks both H3K4me3 and H3K79me2 signal, indicating that it is highly unlikely to be an alternative promoter; and **(d) **La-related protein 1 (*LARP1*) - a BEST locus (black dashed box) positioned between the annotated promoter (red dashed box) and the likely active promoter (green black box) lacks both H3K4me3 and H3K79me2 peaks, indicating that it is highly unlikely to be an alternative promoter.

We next sought to use GRO-seq data aligning to the promoter regions of each of the active genes to train a probabilistic model (NBC) for genome-wide prediction of BEST events (Materials and methods). Specifically, for each of three categories of interest (non-transcribed, BEST, transcriptional elongation), we computed the probability distributions for six distinct features (Figure 2). Then for every non-promoter-associated 2-kb window across the entire genome, which we defined as any window at least 7 kb away from a known RefSeq transcription start site or IMR90 H3K4me3 peak (Materials and methods), we used these distributions to calculate the probability that it belongs to each of the three categories, and assigned a category based on highest probability (Materials and methods). Using a logarithm of odds (LOD) score threshold of 2.5, we predicted 9,662 high-confidence non-promoter-associated BEST loci (Figure 3a). The widespread occurrence of non-promoter-associated BEST throughout the genome, particularly within intergenic and inactive intragenic regions, indicates that RNAP specifically associates with non-promoter loci.

### Non-promoter-associated BEST loci correlate with open chromatin regions enriched for H3K27ac

Approximately 70% (*n *= 6,770/9,662) of the genome-wide BEST predictions overlap IMR90 open chromatin loci (DHSs; Figure 3a), which represents an approximately nine-fold enrichment over background expectation (Materials and methods). Furthermore, almost 85% (*n *= 5,678/6,770) of these also overlap regions significantly enriched for H3K27ac in IMR90 cells (the most reliable chromatin marker to-date of active enhancers [22]), which represents a striking approximately 30-fold enrichment relative to background (Figure 3a). Manual inspection of several loci confirmed the tendency for non-promoter-associated BEST loci to overlap DHS regions that are enriched for H3K27ac but not for H3K4me3 (Figure 3b-d).

**Figure 4:**
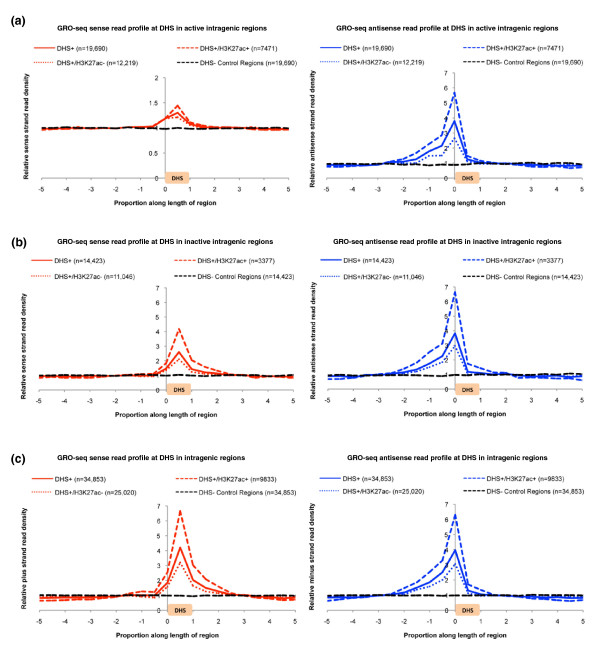
**BEST signature at IMR90 DNase hypersensitive sites and H3K27ac peak regions in IMR90 cells**. **(a-c) **Signal for BEST (accumulation of GRO-seq sense reads accompanied by anti-sense reads immediately upstream) is shown at IMR90 DHSs located within actively transcribed intragenic regions (a), non-transcribed intragenic regions (b), and intergenic regions (c). Relative sense/plus read density (y-axis) is the sense/plus read density at a particular proportional position divided by the average sense/plus read density in the entire DHS + flanking region. Proportional positions between 0 and 1 on the x-axis correspond to the DHS peak. Positions < 0 and > 1 correspond to flanking regions. IMR90 DHSs and H3K37ac peaks potentially associated with promoters or gene ends were discarded from the analysis. Non-DHS control regions (black) were randomly generated and follow the same size distribution as DHSs.

To further characterize BEST at DHS regions, we compared the profiles of GRO-seq sense/anti-sense read density at non-promoter-associated DHS and non-DHS control sites within transcribed intragenic (Figure 4a), non-transcribed intragenic (Figure 4b), and intergenic loci (Figure 4c) (Materials and methods). For all three categories, we observed a significant accumulation of sense reads within DHSs and anti-sense reads immediately upstream (Figure 4a-c) - precisely the signature of BEST. The signal for BEST was even more pronounced at DHSs that overlap H3K27ac peaks (Figure 4a-c). In intragenic loci, the accumulation of GRO-seq anti-sense reads at DHSs appears more pronounced than GRO-seq sense reads (Figure 4a-b); however, this is most likely because we are normalizing read density in a particular window by the average read density in the entire region, and the average read density is always higher in the sense orientation.

### Non-promoter-associated BEST regions are preferentially associated with active enhancers

**Figure 5:**
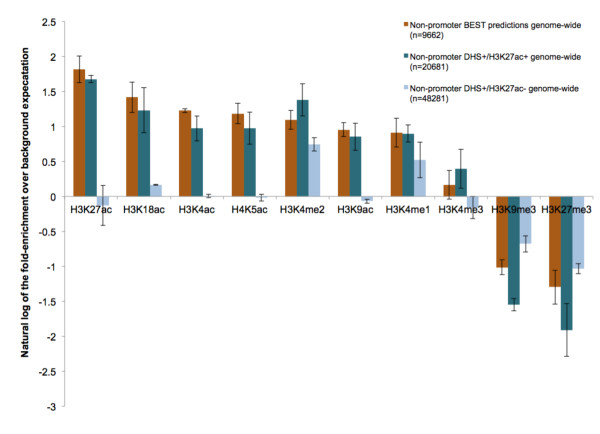
**Relative representation of ten different chromatin marks at predicted BEST loci in IMR90 cells**. The natural logarithm (ln) of the fold-enrichment over background (y-axis) is shown for ten different histone modifications at high-confidence BEST predictions (brown), DHS^+^/H3K27ac^+ ^regions (dark blue), and DHS^+^/H3K27ac^- ^regions (light blue). Background regions are non-promoter, non-DHS, 2-kb windows randomly selected from the genome. Error bars represent the standard deviation among biological replicates.

To analyze the chromatin landscape at the predicted non-promoter-associated BEST loci more comprehensively, we assessed the representation of ten different histone modifications for which IMR90 data from chromatin immunoprecipitation followed by high-throughput sequencing (ChIP-seq) and corresponding control (input) data were available for two samples from the Epigenome Atlas (Materials and methods). The most strongly enriched modification was H3K27ac (Figure 5), which is a robust discriminator between active and poised enhancers [22,23]. The second-most enriched mark was H3K18ac, which is also thought to be associated with functional enhancers [29]. The two least-enriched modifications were H3K4me3, which is associated primarily with promoters, and histone H3 lysine 4 monomethylation (H3K4me1), which is thought to be present at both active and inactive/poised enhancers [29]. Finally, two modifications associated with repressed states [30], histone H3 lysine 27 trimethylation (H3K27me3) and histone H3 lysine 9 trimethylation (H3K9me3), were depleted at regions of BEST.

We repeated the above-described analysis for non-promoter-associated DHSs that overlap H3K27ac peaks (DHS^+^/H3K27ac^+^) and for those that do not (DHS^+^/H3K27ac^-^) (Figure 5). Expectedly, DHS^+^/H3K27ac^+ ^regions were most enriched for H3K27ac and other active chromatin marks, and were depleted for repressive marks (Figure 5). DHS^+^/H3K27ac^- ^regions were depleted for most active and repressive marks, and were enriched only for marks often associated with poised states, H3K4me1 and H3K4me2 (Figure 5). Most importantly, regions of BEST exhibited a chromatin landscape significantly more similar to that of candidate active enhancers (DHS^+^/H3K27ac^+^) than poised enhancers (DHS^+^/H3K27ac^-^).

To confirm this finding in another cell type, we turned to mouse embryonic stem cells (mESCs), which is the only other cell type in which both the nascent transcriptome (GRO-seq) [31] and enhancer-related chromatin marks (ChIP-seq) [22,32-35] have been extensively characterized. We re-trained the NBC using mESC GRO-seq data aligning to active promoters, and applied the NBC genome-wide to predict non-promoter-associated BEST loci. Using a genome-wide dataset of candidate mESC enhancers [35], we found that robustly active enhancers are approximately 8.5-fold enriched (*P *< 0.0001) for BEST relative to poised enhancers (Additional file 3). Furthermore, approximately 71% (*n *= 5/7) of the candidate enhancers that were validated by an *in vitro *reporter gene assay [32] were predicted as BEST loci (Materials and methods). Collectively, these results indicate that BEST regions, as predicted by our classifier, are preferentially associated with active enhancer elements located within both transcribed and non-transcribed genomic regions.

### BEST is robust at CTCF binding sites that overlap H3K27ac peaks

**Figure 6:**
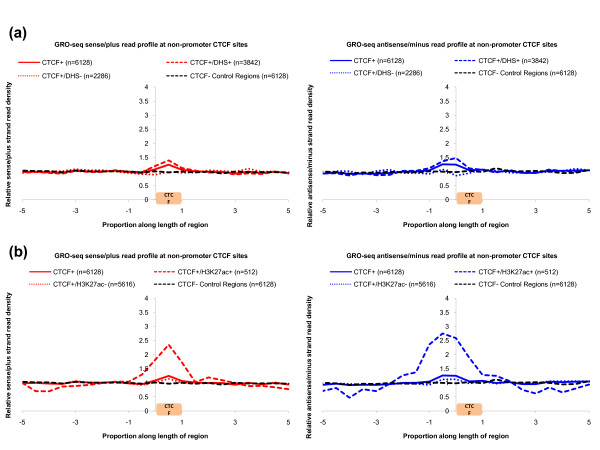
**BEST signature at IMR90 CCCTC-factor binding regions**. **(a,b) **Signal for BEST (accumulation of GRO-seq sense reads accompanied by anti-sense reads immediately upstream) is shown at non-promoter-associated IMR90 CTCF binding regions stratified by open chromatin loci (DHSs) (a) and H3K27ac peaks (b). Relative sense/plus read density (y-axis) is the sense/plus read density at a particular proportional position divided by the average sense/plus read density in the entire CTCF + flanking region. Proportional positions between 0 and 1 on the x-axis correspond to CTCF binding regions [36]. Positions < 0 and > 1 correspond to flanking regions. IMR90 CTCF binding regions potentially associated with promoters or gene ends were discarded from the analysis. Non-CTCF control regions (black) were randomly generated and follow the same size distribution as IMR90 CTCF binding regions.

Distal regulatory elements are not limited to enhancers; another important class is target sites for CTCF, which have many known functions, including insulator activity. To assess BEST at IMR90 CTCF binding sites [36], we followed the same method as for DHSs to assess GRO-seq sense and anti-sense read density profiles (Materials and methods). Relative to non-CTCF control regions, we detected a robust signal for BEST at CTCF binding sites that overlap H3K27ac peaks (CTCF^+^/H3K27ac^+^), only a very modest signal at sites that overlap DHS peaks alone (CTCF^+^/DHS^+^), and no signal at sites that overlap neither (Figure 6a,b). The results are consistent with the previous finding that CTCF can sometimes recruit RNAP to CTCF binding sites [37]. However, the pronounced signal at CTCF^+^/H3K27ac^+ ^sites, together with the dampened signal at CTCF^+^/DHS^+ ^sites, suggests that this recruitment may be most prevalent at CTCF binding sites that function as, or are proximal to, active enhancers.

## Discussion

In this study, we analyzed the IMR90 nascent transcriptome [10], and developed a probabilistic model of promoter-proximal transcriptional pausing in order to identify non-promoter-associated BEST. Further computational analysis, using genome-wide IMR90 chromatin profiles (Epigenome Atlas), revealed that non-promoter BEST is significantly associated with regions enriched for chromatin marks (such as DHSs, H3K27ac and H3K18ac) that demarcate active enhancers.

RNAP pausing is well-appreciated at promoter regions [16,17], and very recently has been discovered at specific loci within actively transcribed genes in yeast [12,19,20]. RNAP pausing has also been observed at cohesin binding sites within a single actively transcribed human gene in human umbilical vein endothelial cells (HUVECs) [38]. However, to the best of our knowledge, our work is the first systematic, genome-scale investigation of non-promoter RNAP pausing in human cells. A recent study identified systematic biases in next-generation sequence data, such that an accumulation of GRO-seq sense reads may not necessarily reflect *bona fide *pausing, due to various nucleotide preferences during cDNA amplification and sequencing [39]. Our analysis circumvents this issue by defining pausing as an accumulation of both sense and anti-sense GRO-seq reads, reflecting the widespread divergent transcription associated with promoter-proximal RNAP pausing in mammalian cells [17].

Recent *in silico *strategies to identify RNAP pausing from nascent RNA sequencing data have used a local, deterministic approach - a minimum level of enrichment of sense read density in a particular window relative to neighboring windows [12,40]. In contrast, our approach utilizes a probabilistic model trained on a reliable genome-wide dataset. The model can be trained on, and applied to, any GRO-seq dataset in order to make inferences about the most likely active enhancer elements. Applying the model to IMR90 GRO-seq data, we detected thousands of non-promoter BEST events. Some of these are located within actively transcribed regions; therefore, the BEST could be due to bound transcription factors that hinder RNAP processivity and induce pausing [41]. However, many of the BEST events are located in intergenic regions. BEST at these loci may be due to specific recruitment of RNAP to active enhancers, as reported previously [42,43]. In fact, a seminal RNA-seq-based study reported that many neuronal enhancers recruit RNAP, which then transcribes bi-directionally a novel class of transcripts termed enhancer RNAs (eRNAs) [43]. A more recent GRO-seq-based study reported significant and dynamic changes to the cellular eRNA profile upon application of an exogenous trigger [40]. It is quite possible that predictions of BEST events using our approach coincide with regions that produce eRNAs as defined by these two studies.

Two major unanswered questions are whether RNAP-mediated BEST occurs at all active enhancers, and whether it contributes to the maintenance of open chromatin, or is just a consequence of the presence of other factors at those sites. A critical related question is whether the short bi-directional transcripts produced by BEST at these sites have functional relevance, or are simply transcriptional noise tolerated by evolution because of relatively minor metabolic cost. Either way, the ability to detect BEST by analysis of GRO-seq data contributes another important approach for the dissection of genomic regulation in higher eukaryotes.

## Conclusions

Long-range regulatory elements are important modulators of gene expression, but they remain poorly annotated. Recent approaches for genome-wide identification of regulatory elements have focused on analyzing the chromatin state. This study contributes an alternative, complementary strategy. We developed a probabilistic model to capture the transcriptomic signature, BEST, of promoter-proximal polymerase pausing. We used this model to predict non-promoter-associated BEST regions, which were significantly enriched for chromatin marks (such as H3K27ac) that are associated with active long-range regulatory elements.

## Materials and methods

### Identifying actively transcribed genes

All human RefSeq transcripts (*n *= 35,983) were downloaded in hg18 coordinates from the UCSC Table Browser, build 36 [44]. Only validated mRNA transcripts ('NM_' prefix) were retained (*n *= 30,326). For each transcript longer than 3 kb (*n *= 27,863), we defined the body of the transcript as 1 kb downstream of the transcription start site to the annotated gene end. The level of transcription for each transcript body in IMR90 cells was determined by computing the average GRO-seq sense reads/kb/mapability using the previously published IMR90 GRO-seq data [10] and the 'Duke Uniq 35' mapability data downloaded from the UCSC Table Browser, build 36. A reads/kb/mapability cutoff for transcriptional activity was chosen according to the maximal accuracy measure - the reads/kb/mapability that achieves the optimal combination of sensitivity (true positive rate) and specificity (true negative rate) using high-confidence true positive genes (expressed genes; *n *= 1,522) and true negative genes (non-expressed genes; *n *= 2,046) from a published IMR90 microarray dataset [26]. Accuracy was measured by calculating the following: (Number of expressed genes identified + Number of non-expressed genes identified)/All genes. At the most accurate cutoff of 5 reads/kb/mapability, 14,145 transcripts were called active, of which 5,213 uniquely mapped to a gene symbol.

### Naïve Bayes classifier for prediction of BEST events

GRO-seq data aligning to (i) active promoters (*n *= 5,213), (ii) active transcript bodies (*n *= 5,213), and (iii) randomly selected intergenic regions (with a similar length distribution as active RefSeq transcripts, but non-overlapping with any known RefSeq transcript; *n *= 5,213) were used to train a NBC to identify BEST, transcriptional elongation, and non-transcribed regions, respectively. First, 2-kb windows were centered at all active start sites of transcription, mid-points of active transcripts, and mid-points of the randomly selected intergenic regions. Then, for all 2-kb test windows in each class, the values for the following six features were calculated: (i) sense strand reads/kb/mapability (hereafter 'read density'), (ii) antisense strand read density, (iii) ratio of the sense strand read density to that in a 2-kb window immediately 3' of the test window, (iv) ratio of the antisense strand read density to that in a 2-kb window immediately 3' of the test window, (v) ratio of the sense strand read density to that in a 2-kb window immediately 5' of the test window, and (vi) ratio of the antisense strand read density to that in a 2-kb window immediately 5' of the test window. The values of these features were used to compute a probability distribution for each feature for each class. These distributions were utilized by the NBC, according to the following equation, to differentiate BEST events from productive transcriptional elongation and transcriptional noise:

$$Class=\underset{c_{i}\in \left\{B,E,N\right\}}{\mathrm{argmax}}\text{Pr}\left(c_{i}\right)\overset{6}{\underset{j=1}{\prod }}\text{Pr}\left(f_{j}\left|c_{i}\right)\right)$$

where the three classes *B, E*, and *N *represent BEST, transcriptional elongation, and non-transcribed, respectively. The prior probabilities, Pr(*c_i_*), were set to be equal for all classes. The six described features are represented by *f_1 _*to *f_6_*. The NBC was applied genome-wide, on both strands, avoiding regions associated with promoters or gene ends (within 7 kb of known transcription start sites, annotated gene ends, and IMR90 H3K4me3 peaks). On the plus strand, plus strand GRO-seq reads are interpreted as 'sense' and minus strand GRO-seq reads are interpreted as 'antisense'; on the minus strand, plus strand GRO-seq reads are interpreted as 'antisense' and minus-strand GRO-seq reads are interpreted as 'sense'. On each strand, for each non-overlapping 2-kb test window in the search space, a LOD score was computed comparing the probability of BEST with that of the other two classes:

$$LOD\left(B\right)=\text{ln}\left(\frac{\text{Pr}\left(B\right)\overset{6}{\underset{j=1}{\prod }}\text{Pr}\left(f_{j}\left|B\right)\right)}{\underset{c_{i}\in \left\{E,N\right\}}{\max}\text{Pr}\left(c_{i}\right)\overset{6}{\underset{j=1}{\prod }}\text{Pr}\left(f_{j}\left|c_{i}\right)\right)}\right)$$

Test windows with LOD scores > 2.5 on one or both strands were set as high-confidence BEST loci.

### Identification of DHS, H3K27ac, and CTCF peaks

IMR90 DNase-seq read data for four biological replicates were downloaded from the Epigenome Atlas, release 3 [45]. MACS [46] version 1.4 was run on each dataset, using the parameter values described previously [47], to identify genomic regions of enrichment for DNase-seq reads. Regions called as enriched in all four replicates were defined as 'DHS peaks'. IMR90 H3K27ac ChIP-seq read data for two biological replicates, and corresponding control (input) data, were downloaded from the Epigenome Atlas, release 3. MACS version 1.4 was run on each dataset, using the default parameter values, to identify genomic regions enriched for H3K27ac. Regions called as enriched in both replicates were defined as 'H3K27ac peaks'. Finally, IMR90 ChIP-chip-derived CTCF peaks were downloaded from the Ren laboratory website [48] and converted to hg18 coordinates using the command line liftOver program with the -minMatch parameter set to 0.9.

### GRO-seq sense and anti-sense read profiling analysis at DHS and CTCF peaks

DHS/CTCF peaks were categorized as located within actively transcribed intragenic regions, inactive intragenic regions, or intergenic regions, with respect to the RefSeq dataset used in this study (see the 'Identifying actively transcribed genes' section of the Materials and methods). To avoid promoter-associated peaks, DHS/CTCF peaks + 5 kb flanking regions that were within 2 kb of known transcription start sites, annotated gene ends, or IMR90 H3K4me3 peaks were discarded. For each of the remaining DHS/CTCF peaks within each category, GRO-seq sense and anti-sense reads/kb/mapability were computed in 150-bp windows from the start of the DHS/CTCF peak to the end of 5-kb flanking regions on either side. Then, for each DHS/CTCF peak and flanking region, nucleotide distance was converted to proportional distance. For example, for a DHS/CTCF peak that is 300 bp in length, the first 150 bp immediately upstream of the peak corresponds to '-0.5 to 0', the first 150 bp within the peak corresponds to '0 to 0.5', the second 150 bp within the peak corresponds to '0.5 to 1', the first 150 bp immediately downstream of the peak corresponds to '1 to 1.5', and so on.

### Representational analysis of chromatin marks at predicted BEST loci

IMR90 ChIP-seq read data for ten different histone modifications, each with at least two biological replicates, and corresponding control (input) data, were downloaded from the Epigenome Atlas, release 3. For each histone modification dataset, the read density (reads/bp) was computed at predicted, high-confidence BEST loci, and then divided by the read density at randomly generated background (control) regions (2 kb in length and drawn from the same genomic locations as BEST loci), to yield an enrichment value. The enrichment value was then divided by the enrichment value for input, to yield a normalized enrichment value.

### Analysis of mouse embryonic stem cell enhancers

To perform genome-wide prediction of BEST loci in an additional cell type, the NBC was trained and applied on publicly available mESC GRO-seq data in exactly the same manner as was done using GRO-seq data from IMR90 cells. Genome-wide candidate mESC enhancers (poised, weak, and strong) were downloaded from Zentner *et al. *[35] and *in vitro *validated mESC enhancers were downloaded from Schnetz *et al. *[32]. In both cases, only those not within 7 kb of known transcription start sites, annotated gene ends, and mESC H3K4me3 peaks were retained for further analysis.

## Abbreviations

BEST: bidirectional expression of short transcripts; ChIP-seq: chromatin immunoprecipitation followed by high-throughput sequencing; CTCF: CCCTC binding factor; DHS: DNase hypersensitive site; eRNA: enhancer RNA; GRO-seq: global nuclear run-on assay followed by high-throughput sequencing; H3K18ac: histone H3 lysine 18 acetylation; H3K27ac: histone H3 lysine 27 acetylation; H3K4me1: histone H3 lysine 4 mono-methylation; H3K4me3: histone H3 lysine 4 tri-methylation; IMR90: human lung fibroblasts; LOD: logarithm of odds; mESC: mouse embryonic stem cell; NBC: Naïve Bayes classifier; RNAP: RNA polymerase.

## Competing interests

The authors declare that they have no competing interests.

## Authors' contributions

PS conceived of, designed, and co-supervised the study, carried out computational analyses, and wrote the manuscript. MFM carried out computational analyses and participated in manuscript preparation. FSC co-supervised the study and edited the manuscript. All authors read and approved the final manuscript.

## Supplementary Material

Additional file 1 **Receiver operating characteristic (ROC) curve depicting the sensitivity and specificity at various IMR90 GRO-seq read density cutoffs for gene activity**. This figure shows that a cutoff of 5 reads/kb/mapability achieves the best combination of sensitivity and specificity, according to the maximal accuracy metric. Click here for file

Additional file 2 **Inverse correlation between promoter-proximal pausing index and level of gene transcription in IMR90 cells**. This figure shows that promoter-proximal pausing of RNA polymerase is high for lowly expressed genes and low for highly expressed genes. Click here for file

Additional file 3 **Representation of BEST at three different enhancer subtypes in mouse embryonic stem cells**. This figure shows that signal for BEST (bidirectional expression of short transcripts) is approximately two-fold and approximately eight-fold enriched at strong enhancers relative to weak enhancers and poised enhancers, respectively. Click here for file

## References

[B1] Maniatis T Reed R An extensive network of coupling among gene expression machines. Nature 2002 416 499 506 10.1038/416499a 11932736

[B2] Komili S Silver PA Coupling and coordination in gene expression processes: a systems biology view. Nat Rev Genet 2008 9 38 48 10.1038/nrg2223 18071322

[B3] Wyrick JJ Young RA Deciphering gene expression regulatory networks. Curr Opin Genet Dev 2002 12 130 136 10.1016/S0959-437X(02)00277-0 11893484

[B4] Kim HD Shay T O'Shea EK Regev A Transcriptional regulatory circuits: predicting numbers from alphabets. Science 2009 325 429 432 19628860 10.1126/science.1171347 PMC2745280

[B5] Hindorff LA Sethupathy P Junkins HA Ramos EM Mehta JP Collins FS Manolio TA Potential etiologic and functional implications of genome-wide association loci for human diseases and traits. Proc Natl Acad Sci USA 2009 106 9362 9367 10.1073/pnas.0903103106 19474294 PMC2687147

[B6] Nicolae DL Gamazon E Zhang W Duan S Dolan ME Cox NJ Trait-associated SNPs are more likely to be eQTLs: annotation to enhance discovery from GWAS. PLoS Genet 2010 6 e1000888 10.1371/journal.pgen.1000888 20369019 PMC2848547

[B7] Nica AC Montgomery SB Dimas AS Stranger BE Beazley C Barroso I Dermitzakis ET Candidate causal regulatory effects by integration of expression QTLs with complex trait genetic associations. PLoS Genet 2010 6 e1000895 10.1371/journal.pgen.1000895 20369022 PMC2848550

[B8] Roeder RG Transcriptional regulation and the role of diverse coactivators in animal cells. FEBS Lett 2005 579 909 915 10.1016/j.febslet.2004.12.007 15680973

[B9] Rougvie AE Lis JT The RNA polymerase II molecule at the 5' end of the uninduced hsp70 gene of D. melanogaster is transcriptionally engaged. Cell 1988 54 795 804 10.1016/S0092-8674(88)91087-2 3136931

[B10] Core LJ Waterfall JJ Lis JT Nascent RNA sequencing reveals widespread pausing and divergent initiation at human promoters. Science 2008 322 1845 1848 10.1126/science.1162228 19056941 PMC2833333

[B11] Nechaev S Fargo DC dos Santos G Liu L Gao Y Adelman K Global analysis of short RNAs reveals widespread promoter-proximal stalling and arrest of Pol II in Drosophila. Science 2010 327 335 338 10.1126/science.1181421 20007866 PMC3435875

[B12] Churchman LS Weissman JS Nascent transcript sequencing visualizes transcription at nucleotide resolution. Nature 2011 469 368 373 10.1038/nature09652 21248844 PMC3880149

[B13] Seila AC Calabrese JM Levine SS Yeo GW Rahl PB Flynn RA Young RA Sharp PA Divergent transcription from active promoters. Science 2008 322 1849 1851 10.1126/science.1162253 19056940 PMC2692996

[B14] Preker P Nielsen J Kammler S Lykke-Andersen S Christensen MS Mapendano CK Schierup MH Jensen TH RNA exosome depletion reveals transcription upstream of active human promoters. Science 2008 322 1851 1854 10.1126/science.1164096 19056938

[B15] Espinosa JM The meaning of pausing. Mol Cell 2010 40 507 508 10.1016/j.molcel.2010.11.025 21095581

[B16] Li J Gilmour DS Promoter proximal pausing and the control of gene expression. Curr Opin Genet Dev 2011 21 231 235 10.1016/j.gde.2011.01.010 21324670 PMC3443551

[B17] Levine M Paused RNA polymerase II as a developmental checkpoint. Cell 2011 145 502 511 10.1016/j.cell.2011.04.021 21565610 PMC4257488

[B18] Gilchrist DA Dos Santos G Fargo DC Xie B Gao Y Li L Adelman K Pausing of RNA polymerase II disrupts DNA-specified nucleosome organization to enable precise gene regulation. Cell 2010 143 540 551 10.1016/j.cell.2010.10.004 21074046 PMC2991113

[B19] Carrillo Oesterreich F Preibisch S Neugebauer KM Global analysis of nascent RNA reveals transcriptional pausing in terminal exons. Mol Cell 2010 40 571 581 10.1016/j.molcel.2010.11.004 21095587

[B20] Alexander RD Innocente SA Barrass JD Beggs JD Splicing-dependent RNA polymerase pausing in yeast. Mol Cell 2010 40 582 593 10.1016/j.molcel.2010.11.005 21095588 PMC3000496

[B21] Song L Crawford GE DNase-seq: a high-resolution technique for mapping active gene regulatory elements across the genome from mammalian cells. Cold Spring Harb Protoc 2010 2010 pdb prot5384 10.1101/pdb.prot5384 PMC3627383 20150147

[B22] Creyghton MP Cheng AW Welstead GG Kooistra T Carey BW Steine EJ Hanna J Lodato MA Frampton GM Sharp PA Boyer LA Young RA Jaenisch R Histone H3K27ac separates active from poised enhancers and predicts developmental state. Proc Natl Acad Sci USA 2010 107 21931 21936 10.1073/pnas.1016071107 21106759 PMC3003124

[B23] Rada-Iglesias A Bajpai R Swigut T Brugmann SA Flynn RA Wysocka J A unique chromatin signature uncovers early developmental enhancers in humans. Nature 2011 470 279 283 10.1038/nature09692 21160473 PMC4445674

[B24] Phillips JE Corces VG CTCF: master weaver of the genome. Cell 2009 137 1194 1211 10.1016/j.cell.2009.06.001 19563753 PMC3040116

[B25] Ohlsson R Lobanenkov V Klenova E Does CTCF mediate between nuclear organization and gene expression?. Bioessays 2010 32 37 50 10.1002/bies.200900118 20020479 PMC6375297

[B26] Kim TH Barrera LO Zheng M Qu C Singer MA Richmond TA Wu Y Green RD Ren B A high-resolution map of active promoters in the human genome. Nature 2005 436 876 880 10.1038/nature03877 15988478 PMC1895599

[B27] Zeitlinger J Stark A Kellis M Hong JW Nechaev S Adelman K Levine M Young RA RNA polymerase stalling at developmental control genes in the Drosophila melanogaster embryo. Nat Genet 2007 39 1512 1516 10.1038/ng.2007.26 17994019 PMC2824921

[B28] Muse GW Gilchrist DA Nechaev S Shah R Parker JS Grissom SF Zeitlinger J Adelman K RNA polymerase is poised for activation across the genome. Nat Genet 2007 39 1507 1511 10.1038/ng.2007.21 17994021 PMC2365887

[B29] Ong CT Corces VG Enhancer function: new insights into the regulation of tissue-specific gene expression. Nat Rev Genet 2011 12 283 293 21358745 10.1038/nrg2957 PMC3175006

[B30] Ernst J Kheradpour P Mikkelsen TS Shoresh N Ward LD Epstein CB Zhang X Wang L Issner R Coyne M Ku M Durham T Kellis M Bernstein BE Mapping and analysis of chromatin state dynamics in nine human cell types. Nature 2011 473 43 49 10.1038/nature09906 21441907 PMC3088773

[B31] Min IM Waterfall JJ Core LJ Munroe RJ Schimenti J Lis JT Regulating RNA polymerase pausing and transcription elongation in embryonic stem cells. Genes Dev 2011 25 742 754 10.1101/gad.2005511 21460038 PMC3070936

[B32] Schnetz MP Bartels CF Shastri K Balasubramanian D Zentner GE Balaji R Zhang X Song L Wang Z Laframboise T Crawford GE Scacheri PC Genomic distribution of CHD7 on chromatin tracks H3K4 methylation patterns. Genome Res 2009 19 590 601 10.1101/gr.086983.108 19251738 PMC2665778

[B33] Chen X Xu H Yuan P Fang F Huss M Vega VB Wong E Orlov YL Zhang W Jiang J Loh YH Yeo HC Yeo ZX Narang V Govindarajan KR Leong B Shahab A Ruan Y Bourque G Sung WK Clarke ND Wei CL Ng HH Integration of external signaling pathways with the core transcriptional network in embryonic stem cells. Cell 2008 133 1106 1117 10.1016/j.cell.2008.04.043 18555785

[B34] Mikkelsen TS Ku M Jaffe DB Issac B Lieberman E Giannoukos G Alvarez P Brockman W Kim TK Koche RP Lee W Mendenhall E O'Donovan A Presser A Russ C Xie X Meissner A Wernig M Jaenisch R Nusbaum C Lander ES Bernstein BE Genome-wide maps of chromatin state in pluripotent and lineage-committed cells. Nature 2007 448 553 560 10.1038/nature06008 17603471 PMC2921165

[B35] Zentner GE Tesar PJ Scacheri PC Epigenetic signatures distinguish multiple classes of enhancers with distinct cellular functions. Genome Res 2011 21 1273 1283 10.1101/gr.122382.111 21632746 PMC3149494

[B36] Kim TH Abdullaev ZK Smith AD Ching KA Loukinov DI Green RD Zhang MQ Lobanenkov VV Ren B Analysis of the vertebrate insulator protein CTCF-binding sites in the human genome. Cell 2007 128 1231 1245 10.1016/j.cell.2006.12.048 17382889 PMC2572726

[B37] Chernukhin I Shamsuddin S Kang SY Bergström R Kwon YW Yu W Whitehead J Mukhopadhyay R Docquier F Farrar D Morrison I Vigneron M Wu SY Chiang CM Loukinov D Lobanenkov V Ohlsson R Klenova E CTCF interacts with and recruits the largest subunit of RNA polymerase II to CTCF target sites genome-wide. Mol Cell Biol 2007 27 1631 1648 10.1128/MCB.01993-06 17210645 PMC1820452

[B38] Wada Y Ohta Y Xu M Tsutsumi S Minami T Inoue K Komura D Kitakami J Oshida N Papantonis A Izumi A Kobayashi M Meguro H Kanki Y Mimura I Yamamoto K Mataki C Hamakubo T Shirahige K Aburatani H Kimura H Kodama T Cook PR Ihara S A wave of nascent transcription on activated human genes. Proc Natl Acad Sci USA 2009 106 18357 18361 10.1073/pnas.0902573106 19826084 PMC2761237

[B39] Schwartz S Oren R Ast G Detection and removal of biases in the analysis of next-generation sequencing reads. PLoS One 2011 6 e16685 10.1371/journal.pone.0016685 21304912 PMC3031631

[B40] Wang D Garcia-Bassets I Benner C Li W Su X Zhou Y Qiu J Liu W Kaikkonen MU Ohgi KA Glass CK Rosenfeld MG Fu XD Reprogramming transcription by distinct classes of enhancers functionally defined by eRNA. Nature 2011 474 390 394 10.1038/nature10006 21572438 PMC3117022

[B41] Palmer AC Egan JB Shearwin KE Transcriptional interference by RNA polymerase pausing and dislodgement of transcription factors. Transcr 2011 2 9 14 10.4161/trns.2.1.13511 PMC3023640 21326903

[B42] De Santa F Barozzi I Mietton F Ghisletti S Polletti S Tusi BK Muller H Ragoussis J Wei CL Natoli G A large fraction of extragenic RNA pol II transcription sites overlap enhancers. PLoS Biol 2010 8 e1000384 10.1371/journal.pbio.1000384 20485488 PMC2867938

[B43] Kim TK Hemberg M Gray JM Costa AM Bear DM Wu J Harmin DA Laptewicz M Barbara-Haley K Kuersten S Markenscoff-Papadimitriou E Kuhl D Bito H Worley PF Kreiman G Greenberg ME Widespread transcription at neuronal activity-regulated enhancers. Nature 2010 465 182 187 10.1038/nature09033 20393465 PMC3020079

[B44] UCSC Table Browser. http://genome.ucsc.edu/cgi-bin/hgTables

[B45] Human Epigenome Atlas. http://www.genboree.org/EdaccData/Release-3/

[B46] Zhang Y Liu T Meyer CA Eeckhoute J Johnson DS Bernstein BE Nusbaum C Myers RM Brown M Li W Liu XS Model-based analysis of ChIP-Seq (MACS). Genome Biol 2008 9 R137 10.1186/gb-2008-9-9-r137 18798982 PMC2592715

[B47] Stitzel ML Sethupathy P Pearson DS Chines PS Song L Erdos MR Welch R Parker SC Boyle AP Scott LJ NISC Comparative Sequencing Program Margulies EH Boehnke M Furey TS Crawford GE Collins FS Global epigenomic analysis of primary human pancreatic islets provides insights into type 2 diabetes susceptibility loci. Cell Metab 2010 12 443 455 10.1016/j.cmet.2010.09.012 21035756 PMC3026436

[B48] IMR90 CTCF ChIP-chip Data. http://licr-renlab.ucsd.edu/download.html

