# Supplementary material for: Discovery of active enhancers through bidirectional expression of short transcripts
Source: Genome Biol. 2011 Nov 14;12(11):R113. doi: 10.1186/gb-2011-12-11-r113 (PMC3334599; doi:10.1186/gb-2011-12-11-r113)
Supplement: Additional file 1 — Receiver operating characteristic (ROC) curve depicting the sensitivity and specificity at various IMR90 GRO-seq read density cutoffs for gene activity. This figure shows that a cutoff of 5 reads/kb/mapability achieves the best combination of sensitivity and specificity, according to the maximal accuracy metric. [file gb-2011-12-11-r113-S1.DOC]

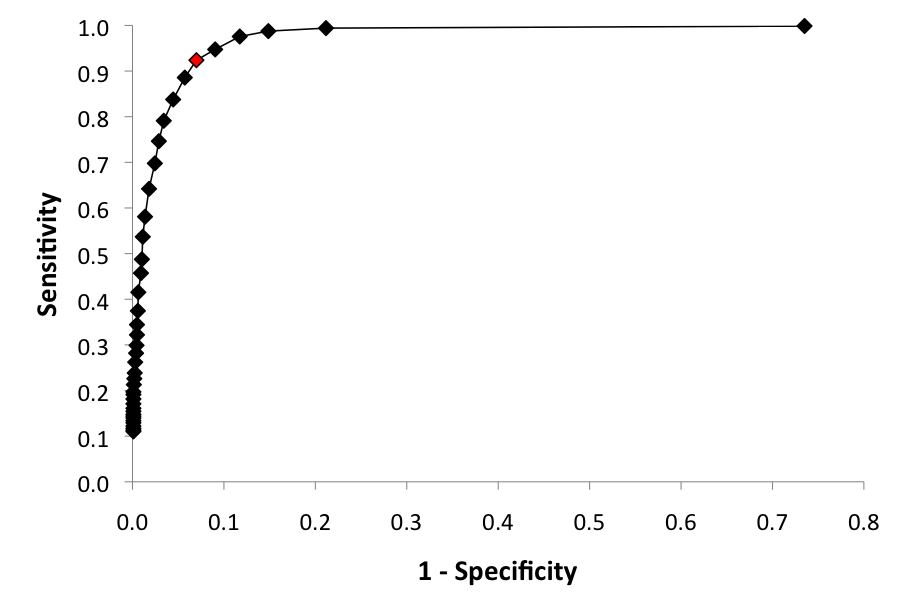


**Figure S1. ROC curve depicting the sensitivity and specificity at various GRO-seq read density cutoffs for gene activity.** Sensitivity (true positive rate) and specificity (true negative rate) are calculated using high-confidence true positive genes (expressed genes; n=1,522) and true negative genes (non-expressed genes; n=2,046) from a published IMR90 microarray dataset^26^. The data point colored in red represents 5 reads/kb/mapability, which achieves the best combination of sensitivity and specificity, according to the maximal accuracy metric (Methods).
