# Supplementary material for: Discovery of active enhancers through bidirectional expression of short transcripts
Source: Genome Biol. 2011 Nov 14;12(11):R113. doi: 10.1186/gb-2011-12-11-r113 (PMC3334599; doi:10.1186/gb-2011-12-11-r113)
Supplement: Additional file 2 — Inverse correlation between promoter-proximal pausing index and level of gene transcription in IMR90 cells. This figure shows that promoter-proximal pausing of RNA polymerase is high for lowly expressed genes and low for highly expressed genes. [file gb-2011-12-11-r113-S2.DOC]

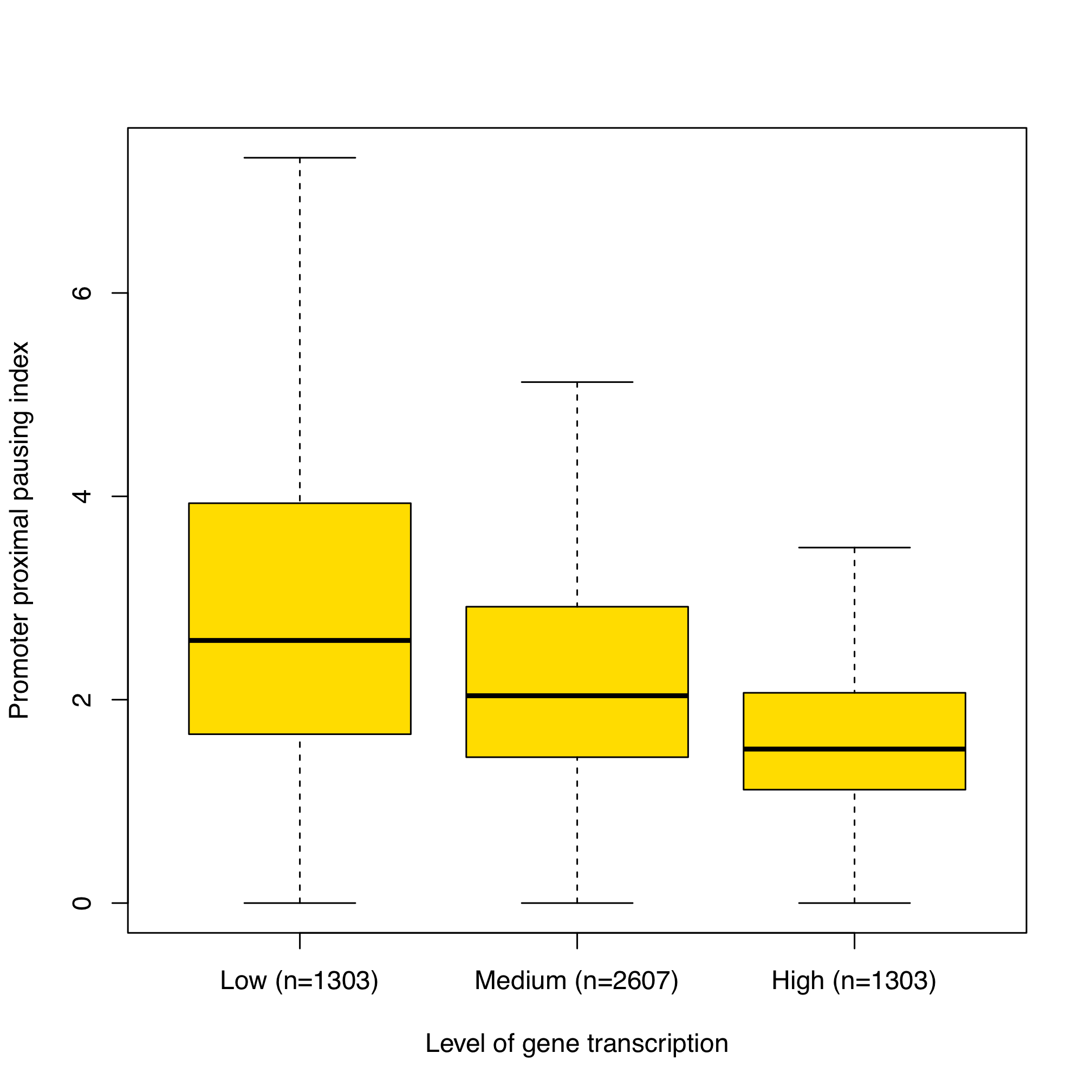


**Figure S2. Inverse correlation between promoter-proximal pausing index and level of gene transcription.** Promoter-proximal pausing index (y-axis) is defined as previously^27,28^. “Low” corresponds to the bottom 25^th^ percentile, “Medium” corresponds to the middle 50^th^ percentile, and “High” corresponds to the top 25^th^ percentile among actively transcribed genes (n=5,213).
