# Supplementary material for: Discovery of active enhancers through bidirectional expression of short transcripts
Source: Genome Biol. 2011 Nov 14;12(11):R113. doi: 10.1186/gb-2011-12-11-r113 (PMC3334599; doi:10.1186/gb-2011-12-11-r113)
Supplement: Additional file 3 — Representation of BEST at three different enhancer subtypes in mouse embryonic stem cells. This figure shows that signal for BEST (bidirectional expression of short transcripts) is approximately two-fold and approximately eight-fold enriched at strong enhancers relative to weak enhancers and poised enhancers, respectively. [file gb-2011-12-11-r113-S3.DOC]

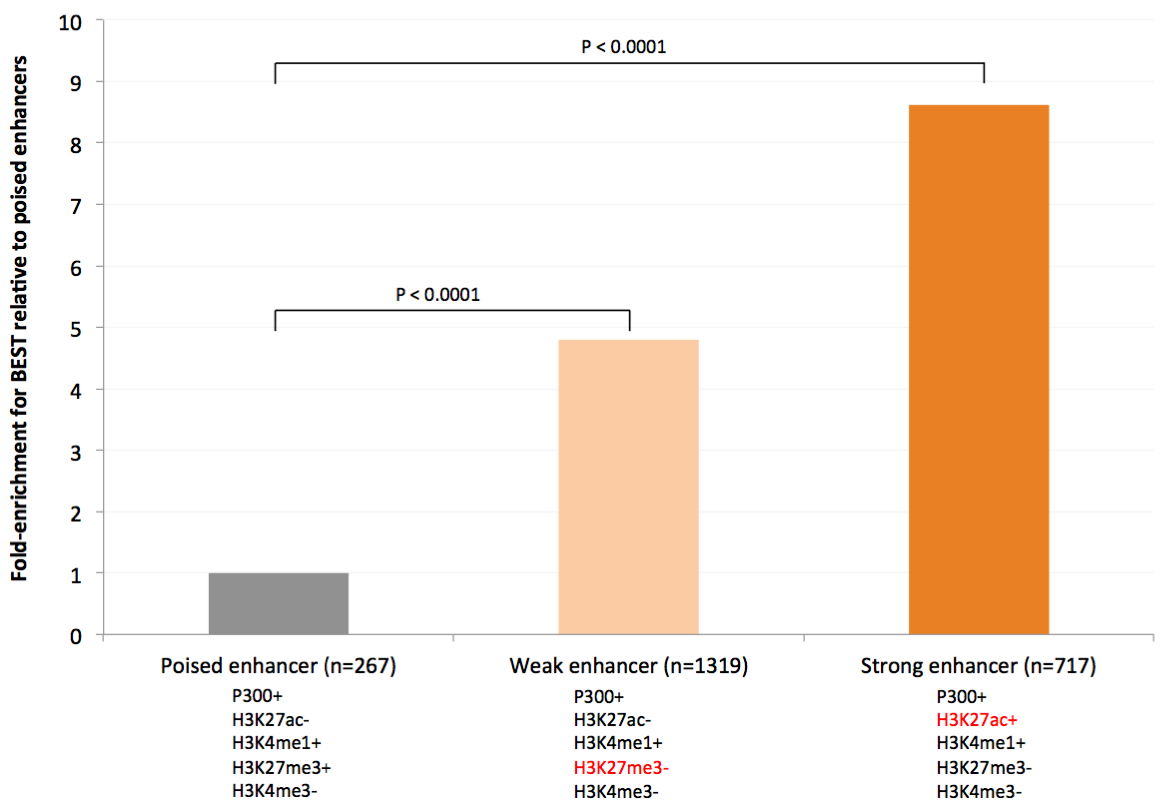


**Figure S3. Representation of BEST at three different enhancer subtypes in mouse embryonic stem cells.** Fold-enrichment for BEST (y-axis) is shown for poised (gray), weakly active (beige), and strongly active (orange) candidate enhancers, as defined by Zentner et al.
